# A geometrical approach explains Lake Ball (Marimo) formations in the green alga, *Aegagropila linnaei*

**DOI:** 10.1038/srep03761

**Published:** 2014-01-20

**Authors:** Tatsuya Togashi, Hironobu Sasaki, Jin Yoshimura

**Affiliations:** 1 Marine Biosystems Research Center, Chiba University, Kamogawa 299-5502, Japan; 2 Department of Mathematics and Informatics, Faculty of Science, Chiba University, Chiba, 263-8522, Japan; 3 Graduate School of Science and Technology and Department of Mathematical Systems Engineering, Shizuoka University, Hamamatsu 432-8561, Japan; 4 Department of Environmental and Forest Biology, State University of New York College of Environmental Science and Forestry, Syracuse, NY 13210, USA

## Abstract

An extremely rare alga, *Aegagropila linnaei*, is known for its beautiful spherical filamentous aggregations called Lake Ball (Marimo). It has long been a mystery in biology as to why this species forms 3D ball-like aggregations. This alga also forms two-dimensional mat-like aggregations. Here we show that forming ball-like aggregations is an adaptive strategy to increase biomass in the extremely limited environments suitable for growth of this alga. We estimate the maximum biomass attained by ball colonies and compare it to that attained by mat colonies. As a result, a ball colony can become larger in areal biomass than the mat colony. In the two large ball colonies studied so far, they actually have larger biomasses than the mat colonies. The uniqueness of Lake Balls in nature seems to be due to the rarity of such environmental conditions. This implies that the conservation of this alga is difficult, but important.

A*egagropila linnaei* (Cladophorales) is a filamentous multicellular freshwater green alga. The detailed life history has not been revealed. The recent phylogenetic analyses based on 18S rRNA gene sequences suggest that freshwater Cladophorales species have adapted from a marine habitat independently at least in the two lineages^1^. *A. linnaei* has an important status as a special natural monument in Japan, is strictly protected as an endangered species in Lake Myvatn, Iceland as well as Lake Akan, Japan^1^,^2^,^3^. Both lakes are freshwater, shallow, naturally eutrophic, and are situated at higher latitudes in the northern hemisphere^2^,^4^,^5^. *A. linnaei* forms spherical, filamentous aggregations of colonies in these lakes known as Lake Ball in English and Marimo in Japanese. However, virtually nothing is known about why they form ball-like aggregations, which may be a key to the conservation of this very rare species.

Field studies of *A. linnaei* have yielded three important observations. First, the ball-like aggregations can grow up to 40 cm in diameter in nature^6^. Large balls (>10 cm in diameter) have a hollow in the centre^1^, because the thickness of the alga is limited to 5 cm from its surface, for photosynthesis^7^. Second, the ball-like aggregations usually form closely packed colonies in a well-defined patch on the bottom of the lake^3^ (see also Fig. 1). Several layers (of smaller balls) are sometimes formed reaching 50 cm in depth^7^. Third, they also form two-dimensional mat-like aggregations on the bottom of the lakes^2^,^8^. Ball-like aggregations and mat-like ones are the same species: it has been confirmed that small pieces of filaments can form either ones^2^,^8^.

Some possible benefits of the ball-like shape have been proposed for this alga. First, ball-like aggregations might be useful to protect the alga from damages caused by fish: free-floating tuft-like filaments of Cladophorales green algae are known to be eaten by rainbow trout in Gannison River, Colorado, USA^6^. However, it appears that *A. linnaei* is not seriously damaged by the feeding actions of fish in either Lake Akan or Lake Myvatn. Second, the ball-like shape might help the colony roll back into the water and avoid drying out if they are washed up on the shore^7^. Finally, the spherical shape also might help wash off any sediment accumulated on the surface as they are turned around by wave action^3^,^9^, thus allowing for photosynthesis from every surface of the ball.

Here we propose an explanation for the formation of the ball-like aggregations, focusing on the volume (biomass). We show, using geometry, that a ball colony can achieve a higher effective areal biomass than the mat colony under the same environmental condition. We demonstrate this empirically by the field observation that the biomass of the ball colonies in Lake Akan is actually higher than that of the mat colonies. Finally, we discuss why ball-like aggregations are so rare from the perspective of the environmental conditions of the lake.

## Results

**Figure 2:**
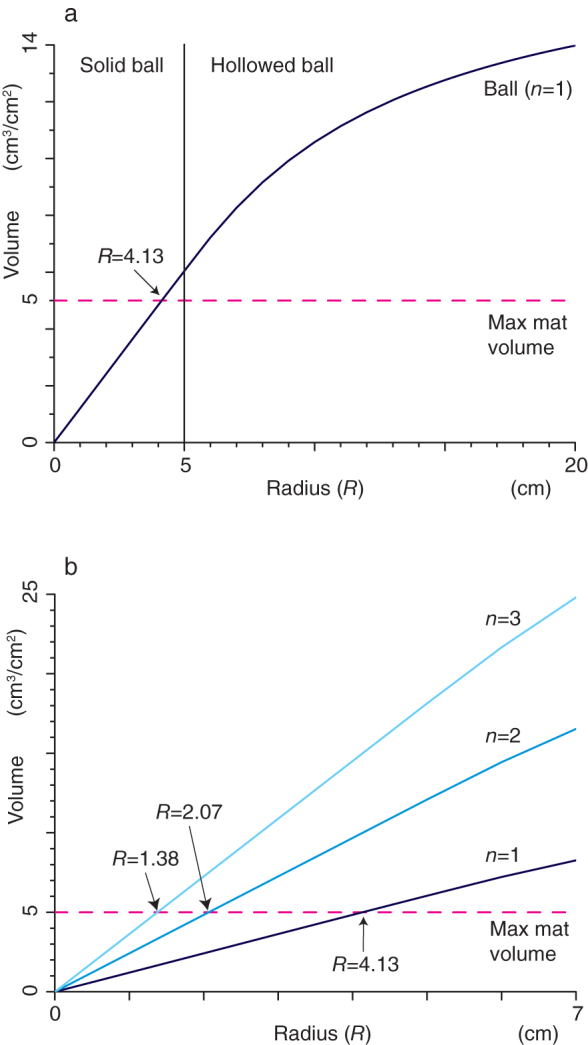
A ball colony can become larger in areal biomass than the mat colony. The biomass (volume) of a closely packed ball colony per unit area plotted against the radius *R* of the ball, when compared to the maximum biomass of the mat colony per unit area. *n* is the number of layers of the ball-like aggregations. (a) The volume of a closely packed ball colony when in a single layer (dark blue line) and the maximum volume of the mat colony (pink dashed line). The minimum radius required for the ball colony to have a greater biomass is 4.13 cm. Lake balls larger than 5 cm in radius have a hollow centre. (b) The volumes of closely packed ball colonies in 1, 2 and 3 layers (dark, medium and light blue lines, respectively) and the maximum volume of the mat colony (pink dashed line). The threshold radii required for a greater biomass are 2.07 and 1.38 cm, respectively, when they are layered two and three deep.

We compare the maximum biomass of the ball and mat colonies to find the optimal solution to maximize the biomass. Fig. 2 shows the comparison between the biomass of a closely-packed ball colony that consists of equal ball-like aggregations with the maximum biomass of the mat colony under the same environmental condition as the ball colony per cm^2^. For a single layer of ball-like aggregations, our analysis shows that the mat colony has a greater biomass if the radius of each ball is under 4.13 cm (Fig. 2a). When the radius increases beyond 4.13 cm, however, the ball colony is predicted to have a greater biomass, and presumably, higher productivity. Note that ball-like aggregations become hollow if the radius is larger than 5 cm. When the number of layers (*n*) is increased, this threshold radius for greater biomass becomes smaller (Fig. 2b). For example, it is 2.07 cm for two layers and 1.38 cm for three layers. This means that two or more layers are necessary to have a greater biomass for the ball colony that consists of solid ball-like aggregations with much less than 5 cm in radius.

**Figure 3:**
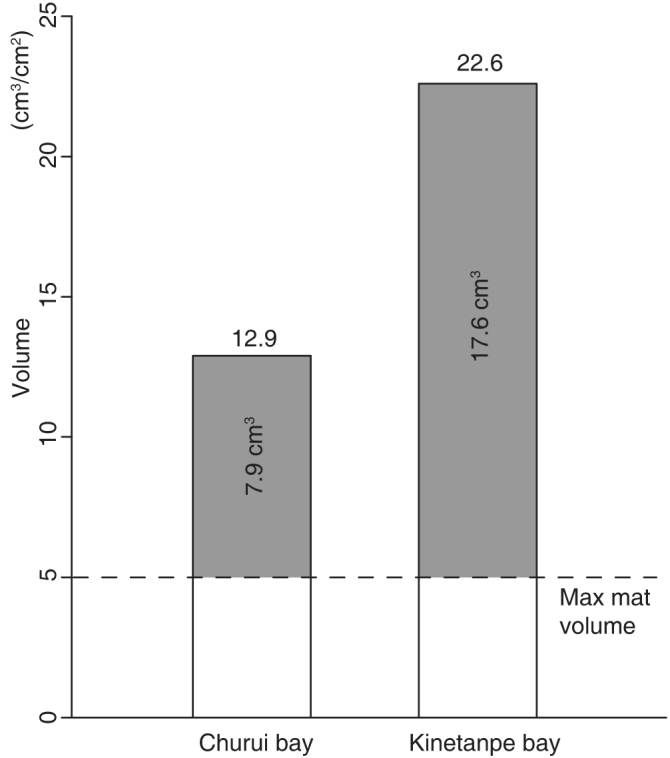
The two large stable ball colonies in Lake Akan actually have larger areal biomasses than the mat colonies. The observed biomass (volume) of ball-like aggregations per unit area in two stable ball colonies in the Churui and Kinetanpe bays in Lake Akan (43°27′N, 144°06′E, eastern Hokkaido, Japan). The increase over the mat colonies is shown in grey. The size classes and numbers of ball-like aggregations in Churui bay are 4.23 × 10^8^ (*d* = 2.5 cm), 0.28 × 10^8^ (*d* = 7.5 cm), 18.9 × 10^4^ (*d* = 12.5 cm), 8.6 × 10^4^ (*d* = 17.5 cm), 1.6 × 10^4^ (*d* = 22.5 cm), 0.3 × 10^4^ (*d* = 27.5 cm) where *d* is the median of diameter of a ball-like aggregation. Similarly, in Kinetanpe bay, they are 2.19 × 10^8^ (*d* = 2.5 cm), 0.35 × 10^8^ (*d* = 7.5 cm), 43.4 × 10^4^ (*d* = 12.5 cm), 12.8 × 10^4^ (*d* = 17.5 cm). The volume of ball-like aggregations at each class is estimated as follows: (4/3)π(*d*/2)^3^*N* without a hollow (*d*/2 ≤ 5) and (4/3)*π*[(*d*/2)^3^ − (*d*/2 − 5)^3^]*N* with a hollow (*d*/2 > 5) where *N* is the number of ball-like aggregations in the class.

We analyse the field data on size classes of ball-like aggregations, the number of ball-like aggregations in each class and the total area of the colonies in two stable Lake Ball congregations in Churui bay and Kinetanpe bay of Lake Akan^10^. Using these data, we estimated the biomass of a ball colony and the maximum biomass of the mat colony that has the same area under the same environmental condition as the ball colony. Note that the maximum biomass of a mat colony is always 5 cm^3^/cm^2^ because the maximum thickness of aggregations of filaments is 5 cm, as described in the introduction. Fig. 3 compares the biomass between a ball colony and the mat colony. In Churui bay, the total biomass of the ball colony is 10.2 × 10^9^ cm^3^ and the maximum total biomass of the mat colony is 3.96 × 10^9^ cm^3^. Similarly, in Kinetanpe bay, the total biomass of the ball colony is 10.3 × 10^9^ cm^3^ and the maximum total biomass of the mat colony is 2.28 × 10^9^ cm^3^. Therefore, in Churui bay, the biomass of the ball colony per cm^2^ is 12.9 cm^3^ because the total area of this colony is 7.92 × 10^8^ cm^2^, implying an increase of 12.9 − 5 = 7.9 cm^3^/cm^2^ by forming ball-like aggregations. Similarly, in Kinetanpe bay, the biomass of the ball colony per cm^2^ is 22.6 cm^3^ because the total area of this colony is 4.56 × 10^8^ cm^2^, implying an increase of 22.6 − 5 = 17.6 cm^3^/cm^2^ by forming ball-like aggregations.

## Discussion

*A. linnaei* shows a continuous increase in the biomass of filaments. In many other holocarpic annual algae, all gametophytes and sporophytes disappear once a year after investing all cell materials to produce gametes and zoospores, respectively. In contrast, swimming cells (gametes or zoospores) are rarely produced by this alga, although they have been observed^11^,^12^. Therefore, this alga does not appear to depend much on sexual reproduction by gametes and asexual reproduction by zoospores in nature. Furthermore, this alga is perennial: the filaments do not disappear throughout the year^8^.

A ball colony can have a larger biomass than the mat colony, with the same area under the same environmental condition as the ball colony (Fig. 2). Specifically, closely packed ball colonies always have a larger biomass than the mat colonies when single-layer ball-like aggregations have a radius greater than 4.13 cm (Fig. 2a). When the spheres are not so large, as in the beginning stages of the ball formation, ball colonies can have a larger biomass than the mat colonies by piling up ball-like aggregations in layers (Fig. 2b). Note that small ball-like aggregations with a radius less than 5 cm actually have a much higher density than larger ones (see the legend of Fig. 3). Thus, *A. linnaei* can efficiently increase biomass by forming ball-like aggregations. We suggest that this is a significant reason why 1) filaments of this alga form ball-like aggregations, 2) the balls become large, and 3) the density of the spheres is remarkably high in nature. Field data support our theory well (Fig. 3). In the stable ball colonies in Lake Akan (Kinetanpe and Churui bays), the biomass of the ball-like aggregation is much higher than the maximum biomass of the mat colony. *A. linnaei* forms ball-like aggregations only in environments suitable for their growth (see also below for more details), and increases the biomass. In worse conditions, however, they remain a mat-like aggregation. It might be because they cannot increase the biomass even if they form ball-like aggregations (Fig. 2).

Lake balls buried under layers of other balls, and filaments in the dark side can also receive light and survive. First, ball-like aggregations are often nearly strictly spherical in shape (e.g. in Churui bay, the mean ratio of the minor axis to the major axis is 0.9)^13^. As they increase the size, they become more strict spheres. Second, lake balls are buoyant because of oxygen bubbles produced by photosynthesis^7^. They sometimes rise even to the surface of the lake^2^. Finally, wave action positively rotates lake balls^2^. Ball-like aggregations are actually formed in suitable places for their photosynthesis, where there are continuous waves (i.e. in shallow waters, see also below for more details). Thus, lake balls frequently rotate and change their relative positions.

The environmental conditions suitable for a ball colony seem to be actually more severe than those for a mat colony. For example, the ball colonies disappeared before mat colonies in Lake Zeller (Zellersee) in Salzburg, Austria, after the water became polluted^6^. In Lake Akan, while both ball and mat colonies still remain, they are separated^2^. Ball-like aggregations are found in shallow waters (ca. 2 m in depth) near the shore, while the mats are found in deeper waters away from the shore^2^. Therefore, there is greater light available to ball colonies, and thus, for algal growth, than to mat colonies^8^. Finally, ball-like aggregations appear to be formed in oligotrophic waters that spring out from the bottom of the lake^6^. Lake Akan is not a representative oligotrophic lake. However, in the northern part of the lake where there are stable ball colonies, Biochemical Oxygen Demand (BOD) is 0.5–1.0 mg/L and a Total Nitrogen/Total Phosphorus ratio is less than 10 (TN/TP)^14^,^15^.

Forming ball-like aggregations can be a mechanism to maximize the biomass in the very limited conditions suitable for their growth. It is working well in the stable ball colonies. Lake Akan and Lake Myvatn are two of the very few lakes in the world that harbor such habitats as needed for Lake Balls. Therefore, these lakes need to be carefully protected and monitored.

## Methods

We estimate the maximum biomass of the ball and mat colonies. Ball-like aggregations are often nearly spherical in shape^16^ and balls of a similar size tend to aggregate each other due to hydrodynamics^17^. Therefore, our first problem is to determine how to closely pack equal spheres in a unit space. The optimal solution for it is given by the Kepler conjecture in geometry^18^. Our second problem is how to estimate the maximum biomass of the mat colony, with the same area and under the same environmental condition as the ball colony.

**Figure 4:**
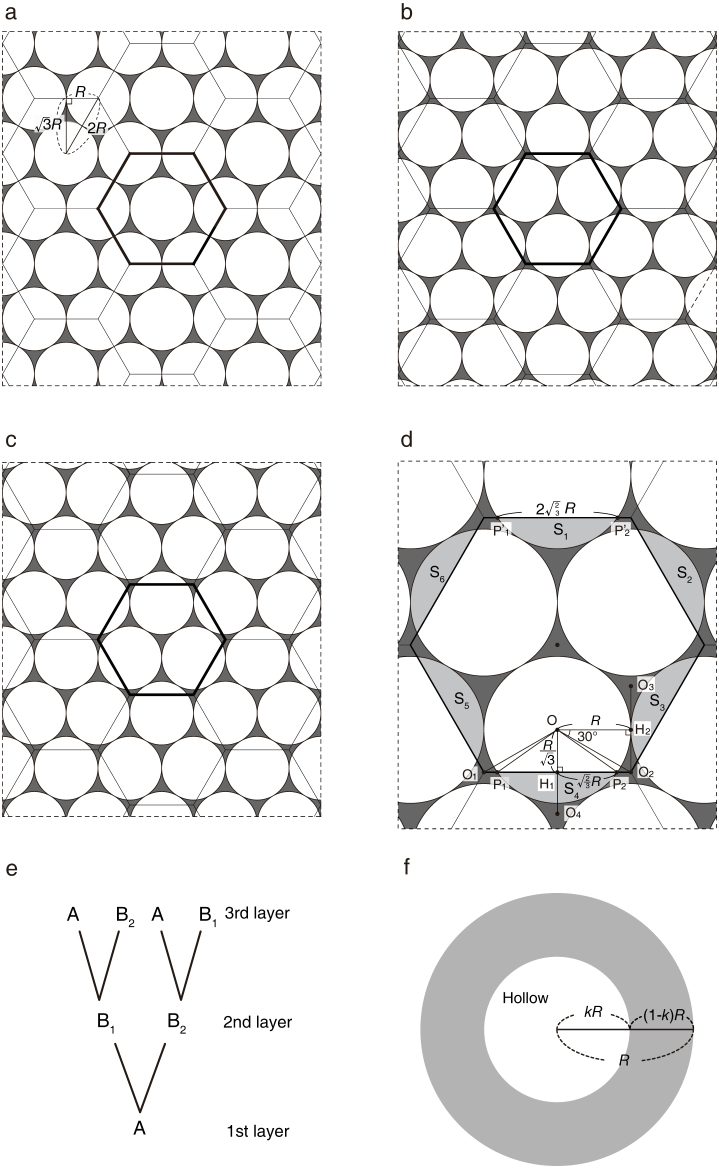
The optimal arrangement of ball-like aggregations with radius *R*. Note that a-e are the plan views of each layer. (a) The hexagonal closely packed structure of the first (bottom) layer (Pattern A) with their measurements. The unit hexagon (within the thick lines) includes the volume of three whole ball-like aggregations (one whole sphere and six one-third spheres). (b) An arrangement of ball-like aggregations in the second layer (Pattern B_1_). (c) Another arrangement of ball-like aggregations in the second layer (Pattern B_2_). (d) Detailed measurements of the unit hexagon with Pattern B_1_, showing that the unit hexagon includes the volume of three ball-like aggregations again. S_1–6_ are the partial spheres. (e) Possible vertical arrangements of the three patterns (A, B_1_ and B_2_) up to three layers. (f) The structure of a ball-like aggregation with a hollow centre, where *k* is the ratio of the radius of the hollow centre to the radius of the entire ball. For details, see text.

First, we consider a case where ball-like aggregations do not have a hollow centre. The optimal arrangement of spheres with the minimal total gap has a hexagonal close-packed structure in three-dimensional Euclidean space as shown in Fig. 4, where *R* is the radius of a sphere. The first layer, on which each sphere is surrounded by six other spheres, can be divided into the unit hexagons (pattern A in Fig. 4a). Basic geometry tells us that the total volume (biomass) over a single unit hexagon on the first layer is given by 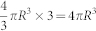. We do not consider the effect of how we treat the edges here, because the total area of a habitat is relatively much larger than the area of the unit hexagon.

The second layer has two mirror-image arrangements depending on where we place a sphere first (pattern B_1_ in Fig. 4b and pattern B_2_ in Fig. 4c). Note that any sphere on this layer is located over the centre of three neighboring spheres on the first layer. Again, in both B_1_ and B_2_ patterns, the biomass over a single unit hexagon on the second layer is always 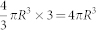. For example, in Pattern B_1_ (Fig. 4b and d), the volume of all partial spheres S_1_ to S_6_ are identical. Therefore, the total volume of S_1_, S_3_ and S_5_ can be replaced by that of S_2_, S_4_ and S_6_.

The overall layer structure becomes as shown in Fig. 4e. If the first layer starts from pattern A, the second layer should be either B_1_ or B_2_. If the second layer is B_1_, the third layer should be either A or B_2_, but, if it is B_2_, that becomes either A or B_1_. As we increase more layers, this structure is maintained. Thus, the total biomass over a single unit hexagon becomes 4*nπR*^3^ where *n* is the number of layers. Because the area of a unit hexagon is given by 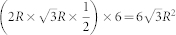, the biomass over the unit area of habitat is 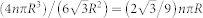.

Second, we consider a case where ball-like aggregations have a hollow centre of radius *kR* where 0 ≤ *k* < 1 (Fig. 4f). Here, because the volume of a hollowed ball-like aggregation is (4/3)*πR*^3^ − (4/3)*πR*^3^*k*^3^ = (4/3)*πR*^3^(1 − *k*^3^), the biomass over a single unit hexagon on each layer is always 4*πR*^3^(1 − *k*^3^). Therefore, the biomass over the unit area is 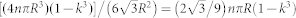. The maximum algal thickness in a ball-like aggregation has been estimated as ca. 5 cm in Lake Akan^2^. As ball-like aggregations become larger (*R* > 5), they have a hollow centre of radius *kR*. Thus, (1 − *k*)*R* = 5, i.e. 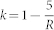.

Finally, we estimate the maximum biomass of a mat colony. The maximum thickness of a mat colony should be approximately equal to the algal thickness in a hollow lake ball, (1 − *k*)*R*, under the same environmental condition, because this thickness would depend on the minimum amount of light necessary for algal photosynthesis. Thus, the maximum biomass of a mat colony also becomes (1 − *k*)*R* = 5 cm^3^/cm^2^. Note that the maximum thickness seen in mat colonies in nature would be different from 5 cm because as we described in the discussion ball and mat colonies are separated under different environmental conditions.

## Author Contributions

T.T. designed the project. T.T. and H.S. performed the research. T.T. analysed the data. T.T. and J.Y. wrote the manuscript.

## Figures and Tables

**Figure 1:**
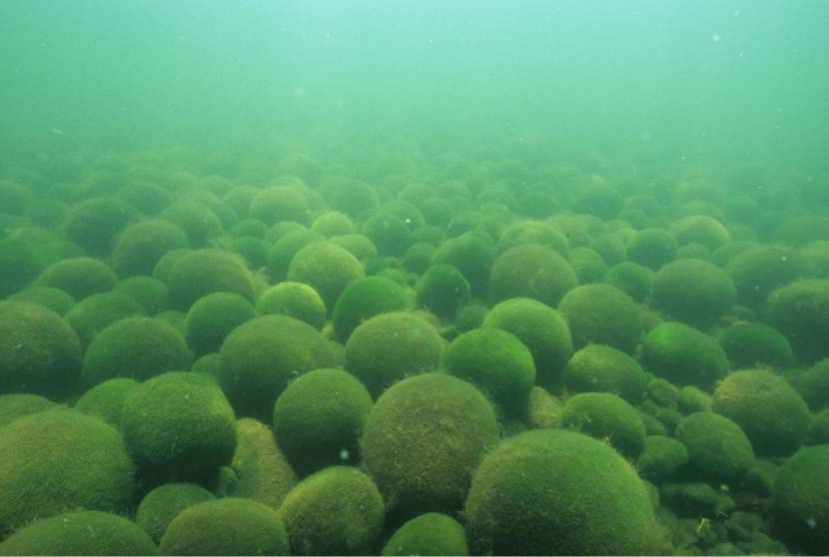
Ball-like aggregations of a similar size form closely packed colonies in a well-defined patch on the bottom of the lake. An overview of Lake Balls (*Aegagropila linnaei*) in Lake Akan (photo by I. Wakana).
